# Editorial: Plant-soil-microbial interactions in arid areas

**DOI:** 10.3389/fpls.2025.1704520

**Published:** 2025-10-21

**Authors:** Huimin Wang, Lu Gong, Xue Wu, Yanju Liu, Xiaodong Yang

**Affiliations:** ^1^ Department of Geography & Spatial Information, Ningbo University, Ningbo, China; ^2^ Institute of Ecology and Environment, Xinjiang University, Urumqi, China; ^3^ Global Centre for Environmental Remediation (GCER), The University of Newcastle (UON), Newcastle, NSW, Australia

**Keywords:** drought stress, environmental filtering, microbial community assembly, plant stress resistance, stochastic processes

Arid and semi-arid regions account for approximately 40-42% of the Earth’s terrestrial surface and represent a critical component of global ecosystems (Lewin et al., 2024). Under long-term water and nutrient limitations, these regions have shaped a large number of species adapted to drought, salinity, and nutrient-poor conditions, which maintain ecosystem stability through deep root systems, leaf morphological traits, and osmotic regulation mechanisms (Schenk and Jackson, 2002). Meanwhile, soils in arid regions serve as important reservoirs of carbon and nutrients, and their physicochemical properties and microbial activities exert profound effects on plant survival and ecosystem processes (Schimel et al., 2007). With the increasing frequency of drought events and the intensification of environmental stresses (IPCC, 2021), elucidating the mechanisms of plant-soil-microbe interactions has become a central scientific issue in arid ecology. In this Research Topic, we have collected 12 articles focusing on plant-soil-microbial interactions in arid regions. These studies include four major aspects: the physiological and chemical mechanisms underlying plant-soil-microbial interactions, microbial community assembly, the regulation of nutrient and carbon cycling mediated by plant-soil-microbial interactions, and the application of these interactions in agriculture and ecosystem restoration. By integrating these findings, this Research Topic delineates a clear scientific trajectory: from elucidating the fundamental mechanisms of plant-soil-microbe interactions, to revealing the patterns of microbial community assembly, and finally to their applications in agriculture and ecological restoration. These studies not only advance our understanding of ecosystem functioning in arid regions but also provide a scientific foundation and practical guidance for the sustainable use and management of these vulnerable ecosystems in the future.

In addition to water scarcity, plants in arid regions are also subjected to multiple other limiting stresses, such as salinity and phosphorus deficiency (Tian et al.; Wang et al.). Under these adverse conditions, soil microorganisms play a critical role in supporting plant survival and growth through various mechanisms. For example, under drought stress, Streptomycetales alleviate nutrient limitations caused by reduced enzymatic activity in plants through the 1-aminocyclopropane-1-carboxylate (ACC) deaminase and the production of secondary metabolites such as indole-3-acetic acid (IAA), thereby enhancing nutrient absorption by sugarcane roots. Rhizobiales counteract drought stress by supporting nitrogen acquisition in sugarcane and producing siderophores to facilitate iron uptake. The synergistic effect of these microorganisms collectively enhances the drought tolerance of sugarcane (Chen et al.). Furthermore, Pan et al. found that inoculation with arbuscular mycorrhizal fungi (AMF) could alleviate manganese stress on *Lespedeza davidii*. Specifically, AMF enhance plant stress resistance by increasing the content of osmotic adjustment substances (e.g., soluble sugars, soluble proteins, free proline), enhancing antioxidant enzyme activities, and reducing membrane lipid peroxidation products, thereby strengthening plant stress tolerance. These studies provide important theoretical support for a deeper understanding of plant-soil-microbe interactions in arid ecosystems.

Microbial community assembly represents a central regulatory element in plant-soil-microbe interactions, providing a foundation for understanding these complex relationships. To clarify microbial community assembly, researchers have conducted extensive experimental studies across different contexts, including artificial vegetation restoration sequences, drought stress gradients, and comparisons between rhizosphere and bulk soils. The results show that stochastic processes (such as dispersal limitation and ecological drift) play an important role in microbial community assembly. By analyzing the microbial communities in the root endosphere, rhizosphere, and bulk soil across three seasons, Zhang et al. found that microbial community assembly was dominated by stochastic processes, with dispersal limitation being the core stochastic process; only bacteria and fungi in bulk soil were regulated by deterministic processes (homogeneous selection) in some seasons. Similarly, in studies on *Pyrus betulifolia*, Liu et al. (2025) further confirmed that both the rhizosphere and endosphere fungal communities were dominated by stochastic processes, and the proportion of these processes in the endosphere was significantly higher than in the rhizosphere. This difference can be attributed to the more enclosed environment of the endosphere and the smaller size of its microbial species pool, which amplifies the influence of ecological drift on community assembly. However, in addition to stochastic processes, environmental filtering (such as the selective effects of factors like soil pH, salinity, nutrients, and moisture) also plays a crucial role in the community assembly. For example,Xue et al. found that dispersal limitation and homogeneous selection dominated the assembly process, with their relative contributions changing dynamically with the duration of artificial vegetation restoration. Among these factors, soil texture and phosphorus were identified as key drivers. These findings provide strong evidence for the crucial role of stochastic processes in microbial community assembly in arid regions.

Plant-soil-microbial interactions play a significant role in nutrient and carbon cycling in terrestrial ecosystems. Root exudates, the functional regulation of microbial communities, and their interactions with soil collectively determine ecosystem productivity, nutrient cycling, and energy flow. Wu et al. , using *Haloxylon ammodendron* and *Haloxylon persicum* as study species, demonstrated that these plants actively shape the structure and function of their rhizosphere microbial communities through root exudates, including triterpenes and fatty acids, which act as key signaling molecules. The enriched beneficial microbes, such as Actinomycetales and Proteobacteria, participate in and drive soil carbon and nutrient cycling. The study by Wang et al. elucidated the regulatory role of plant-soil-microbial interactions in carbon cycling. In the desert-oasis ecotone, microbial community structures differ significantly between rhizosphere and bulk soils. In rhizosphere soil, Proteobacteria and Actinobacteria are relatively abundant; these microorganisms promote heterotrophic respiration by accelerating the degradation of root exudates and the turnover of organic carbon. In contrast, bulk soils are mainly dominated by Actinobacteria, which maintain basal respiration by decomposing recalcitrant soil organic carbon. Luo et al. found that desert shrubs adapt to nutrient-poor environments by differentially regulating root nutrient resorption efficiencies for nitrogen (N), phosphorus (P), and potassium (K). Overall, these plants exhibit a “prioritized K resorption” pattern. Moreover, C_4_ plants show higher K resorption efficiency, whereas C_3_ plants display higher N and P resorption efficiencies. Additionally, plants dynamically adjust their nutrient resorption efficiencies in response to changes in soil properties and climatic factors to better adapt to arid and nutrient-poor environments.

Furthermore, elucidating the mechanisms underlying plant-soil-microbe interactions and applying this knowledge to improve soil quality and optimize nutrient management are central goals for achieving sustainable agriculture and effective ecosystem restoration. Li et al. (2025) found that exogenous carbon application can regulate the structure of soil microbial communities and further enhance plant antioxidant systems, thereby alleviating the effects of salt stress on cotton. Under low-phosphorus conditions, silicon addition can promote the secretion of malic and succinic acids from plant roots, enhance acid phosphatase activity, and enrich phosphorus-solubilizing microbes, including *Bradyrhizobium*, *Paenibacillus*, *Bacillus*, and *Fusarium*, thereby forming an effective “organic acid-microbe” phosphorus system and improving plant phosphorus uptake (Jiang et al.). These studies provide important theoretical and practical support for sustainable agricultural development and ecological restoration.

The aforementioned studies have systematically elucidated the important role of plant-soil-microbial interactions in arid-land ecosystems, providing a solid theoretical foundation for improving plant stress resistance and resource use efficiency through microbial strategies. Despite significant progress made in terms of mechanisms and application potential, future research should further explore the interactive effects of multiple stress factors (such as drought, salinization, and heavy metals) in arid regions, as well as the long-term dynamic patterns of plant-soil-microbial interactions. This will contribute to the development of innovative solutions for the sustainable development of arid regions under global climate change.
